# Adult Neurogenesis: Ultrastructure of a Neurogenic Niche and Neurovascular Relationships

**DOI:** 10.1371/journal.pone.0039267

**Published:** 2012-06-19

**Authors:** Paula Grazielle Chaves da Silva, Jeanne L. Benton, Barbara S. Beltz, Silvana Allodi

**Affiliations:** 1 Programa de Neurobiologia, Instituto de Biofísica Carlos Chagas Filho, Universidade Federal do Rio de Janeiro, Rio de Janeiro, Brazil; 2 Programa de Pós-Graduação em Ciências Morfológicas, Instituto de Ciências Biomédicas, Universidade Federal do Rio de Janeiro, Rio de Janeiro, Brazil; 3 Neuroscience Program, Wellesley College, Wellesley, Massachusetts, United States of America; Institut National de la Recherche Agronomique-CNRS UMR6175, France

## Abstract

The first-generation precursors producing adult-born neurons in the crayfish (*Procambarus clarkii*) brain reside in a specialized niche located on the ventral surface of the brain. In the present work, we have explored the organization and ultrastructure of this neurogenic niche, using light-level, confocal and electron microscopic approaches. Our goals were to define characteristics of the niche microenvironment, examine the morphological relationships between the niche and the vasculature and observe specializations at the boundary between the vascular cavity located centrally in the niche. Our results show that the niche is almost fully encapsulated by blood vessels, and that cells in the vasculature come into contact with the niche. This analysis also characterizes the ultrastructure of the cell types in the niche. The Type I niche cells are by far the most numerous, and are the only cell type present superficially in the most ventral cell layers of the niche. More dorsally, Type I cells are intermingled with Types II, III and IV cells, which are observed far less frequently. Type I cells have microvilli on their apical cell surfaces facing the vascular cavity, as well as junctional complexes between adjacent cells, suggesting a role in regulating transport from the blood into the niche cells. These studies demonstrate a close relationship between the neurogenic niche and vascular system in *P. clarkii.* Furthermore, the specializations of niche cells contacting the vascular cavity are also typical of the interface between the blood/cerebrospinal fluid (CSF)-brain barriers of vertebrates, including cells of the subventricular zone (SVZ) producing new olfactory interneurons in mammals. These data indicate that tissues involved in producing adult-born neurons in the crayfish brain use strategies that may reflect fundamental mechanisms preserved in an evolutionarily broad range of species, as proposed previously. The studies described here extend our understanding of neurovascular relationships in the brain of *P. clarkii* by characterizing the organization and ultrastructure of the neurogenic niche and associated vascular tissues.

## Introduction

**Figure 1:**
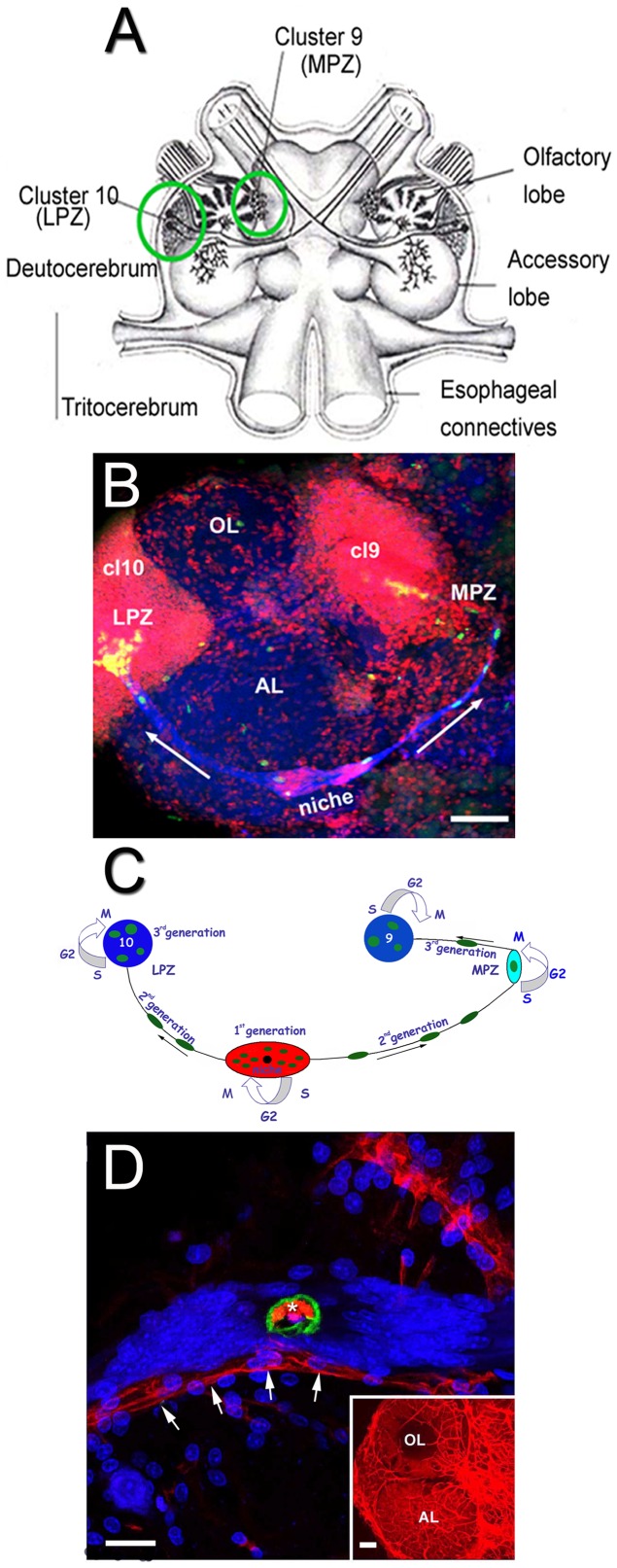
Neurogenic niche in the adult brain of the crayfish *P. clarkii*. (A) Schematic diagram of the crayfish brain showing cell clusters 9 and 10 (circled), and the olfactory and accessory lobes. (B) The lateral proliferation zone (LPZ) in cluster 10 and the medial proliferation zone (MPZ) in cluster 9, connected with each other on the ventral surface of the deutocerebrum by the migratory streams and the neurogenic niche, labeled with an antibody against glutamine synthetase (blue). BrdU (green) labels cells in the niche, streams and proliferation zones. The nuclear marker propidium iodide (red) labels cell body regions, clusters 9 and 10. (C) Model summarizing a view of events leading to the production of new olfactory interneurons in adult crayfish. Neuronal precursor (1^st^-generation) cells reside within a neurogenic niche where they undergo mitosis. Their daughters (2^nd^-generation precursors) migrate along tracts created by the fibers of the niche cells, towards either the lateral proliferation zone (LPZ) or medial proliferation zone (MPZ). At least one more division will occur in the LPZ and MPZ before the progeny (third and subsequent generations) differentiate into neurons. (D) The connection between the cavity in the center of the niche and the vasculature was demonstrated by injecting dextran tetramethylrhodamine (3,000 MW) into the dorsal cerebral artery. The niche sits on top of a blood vessel (arrows). The cavity (asterisk), outlined by anti-Elav (green) labeling, is filled with the dextran dye (red). Propidium iodide (blue) labeling of the nuclei of niche cells is also shown. Inset: shows vasculature of the olfactory (OL) and accessory (AL) lobes labeled with dextran dye. (Images adapted from Beltz et al., 2011). Scale bars: (B) 100 µm; (D) 25 µm; insert in D, 100 µm.

Adult neurogenesis occurs in the brains of phylogenetically diverse species. In higher vertebrates, neural stem cells are located in the subgranular and subventricular zones, where specialized microenvironments maintain and regulate precursor cell lineages producing hippocampal and olfactory bulb neurons, respectively [1], [2]. In the decapod crustacean brain, adult-born neurons are integrated into cell clusters 9 and 10 (terminology of Sandeman et al. [3]), which contain local and projection interneurons that innervate the olfactory and accessory lobes (Figure 1 A). The 1^st^-generation neuronal precursors are found in bilateral neurogenic niches located on the surface of the accessory lobes on the ventral side of the brain (Figure 1 B and C) [4]. The niche precursors divide symmetrically and *both* daughters (2^nd^-generation neuronal precursors) migrate along the processes of the niche cells to proliferation zones in clusters 9 (medial proliferation zone, MPZ) and 10 (lateral proliferation zone, LPZ) [5], [6]. There, they divide at least once more before differentiating into neurons [4].

The 1^st^-generation neuronal precursor cells in *P. clarkii* are unlike the stem cells in the mammalian neurogenic niche, as their geometrically symmetrical divisions are not self-renewing [6]. However, the niche is not depleted as crayfish grow and age [5]. Thus, the neurogenic niche of *P. clarkii* cannot be a closed system; a source external to the niche must provide these neuronal precursors. In vitro experiments show that cells extracted from the hemolymph, and not cells derived from other tissues, are attracted to the niche. It has therefore been proposed that the hematopoietic system may be the source of the neuronal precursors in the crayfish niche [6], [7].

The neurogenic niches in the *P. clarkii* brain are closely associated with the vasculature, as are the niches in the mammalian brain [4], [8], [9], [10]. Dye injections into the dorsal cerebral artery [4], [11] or pericardial sinus [6] in crayfish have revealed that the niche lies on a blood vessel, and that the vasculature communicates with the niche via a central cavity (Figure 1 D); however, the morphological and ultrastructural characteristics of this relationship have not been examined previously. Most cells comprising the crayfish niche are bipolar, each with a short process that terminates on the vascular cavity and a long process that stretches between the niche and either cell cluster 9 or cluster 10 [4]. The long processes of many niche cells fasciculate and form a pathway along which the 2^nd^-generation cells travel (Figure 1 B and C). The niche cells in *P. clarkii*, as well as their processes forming the migratory streams, label with antibodies against glutamine synthetase [4], a marker of glial cells in crustaceans and vertebrates [12], [13], [14], although the sequence of the native molecule is not known (Zhang Y and Beltz BS [2010] Soc Neurosci Abstr 36: 737.14).

Light microscopy of semi-thin sections stained with toluidine blue has previously identified three cell types in the niche. The vast majority (Type I) [5] are the bipolar variety that contain nuclei that are cuboidal or elliptical, with cytoplasmic extensions that contact the rim of the vascular cavity, which is decorated with finger-like cytoplasmic processes. In contrast, Type II and III cells have spherical, densely stained nuclei; Type III cells have an amoeboid shape. It is not known whether these morphologically distinct cell types may be related to each other (e.g, developmental stages of a single cell type), or if they play different roles in the niche. The Type I cells also show striking similarities with cells in the vasculature [5], [15].

In the present study we have explored the ultrastructure of the niche in *P. clarkii* and used light-level, confocal and electron microscopic approaches to examine the relationship between the niche and the underlying vasculature. Our primary goals were to define structural characteristics of the niche microenvironment and to observe specializations at the boundary between the vascular cavity and the niche. Sagittal sections through the vascular cavity and niche show that the niche is a multi-layered structure that is in close contact with blood vessels, and that cells in the vasculature come into contact with the niche. This analysis also defines the ultrastructural characteristics of the three previously identified cell types in the niche, and in addition demonstrates the presence of a fourth morphologically distinct cell type. The bipolar Type I niche cells are by far the most numerous, and are the only cell type present superficially in the most ventral cell layers of the niche. Deeper in the niche (more dorsal), Type I cells are intermingled with Types II, III and IV cells, although these latter cell types are relatively infrequent. Type I cells have dense microvilli, *zonulae adherens* and septate junctions at their apical borders near the vascular cavity, suggesting a role in regulating transport from the blood into the niche. Further, when Type I cells are iontophoresed with Lucifer yellow or dextran-conjugated dyes, these dyes “leak” into the vascular cavity; these findings and the organelles present in these cells indicate a possible secretory function. Features of the cell types and niche morphology are described and discussed, and compared with previously published findings related to the neurogenic niche in the crustacean *Panulirus argus* [10].

The studies described here extend our understanding of neurovascular relationships in the brain of *P. clarkii* by characterizing the organization and ultrastructure of the niche and associated vascular tissues. Further, the specializations of niche cells contacting the vascular cavity are also typical of the interface between the blood/cerebrospinal fluid (CSF)-brain barrier of vertebrates, including cells of the subventricular zone (SVZ) producing new olfactory interneurons in mammals. These ultrastructural data confirm, as proposed previously [4], that tissues involved in producing adult-born neurons in the crayfish brain use strategies resembling those described in the mammalian brain (e.g., [2]), perhaps reflecting fundamental mechanisms that are preserved in an evolutionarily broad range of species.

## Material and Methods

### Animals

Adult male and female freshwater crayfish *Procambarus clarkii* (Malacostraca, Decapoda, Astacidae; carapace length >20 mm for ultrastructural studies; 15–20 mm for immunocytochemical and dye-injection studies), were obtained from Carolina Biological Supply Company (Burlington, NC) and maintained in aquaria with artificial freshwater and a light:dark cycle of 12∶12 hours. Animal care was overseen by full-time staff in the AALAC-approved Animal Care Facility at Wellesley College. All procedures were conducted with a priority on animal welfare.

### Transmission Electron Microscopy (TEM)

The brains of ten animals were dissected in cold crayfish saline (205 mM NaCl, 5.4 mM KCl, 34.4 mM CaCl2, 1.2 mM MgCl2 and 2.4 mM NaHCO_3_). They were then fixed by immersion in 4% paraformaldehyde, 2.5% glutaraldehyde and 0.5% tannic acid in 0.1 M phosphate buffer (PB; pH 7.2) for 2 hours. The samples were postfixed in 1% osmium tetroxide in PB in the dark. Samples were rinsed in PB and stained *en bloc* in 1% uranyl acetate overnight, dehydrated in a graded series of ethanol up to 100% and embedded in Spurr Resin (Electron Microscopy Sciences- Kit #14300). After polymerization for 12 h at 80°C, semithin sections (400–500 nm) were obtained with a Sorvall MT-5000 ultramicrotome (DuPont de Nemours, Wilmington, DE), stained with a mixture of toluidine blue and borax, and examined with a light and fluorescent compound microscope (Zeiss Axioskop 2 microscope equipped with a CCD color camera, Media Cybernetics, Evolution™ MP). Ultrathin sections (60–70 nm) were then obtained from blocks of interest using a RMC MT 6000 ultramicrotome, stained with uranyl acetate and lead citrate, and examined with a JEOL 1200 EX transmission electron microscope (EM) operated at an accelerating voltage of 80 kV.

### Scanning Electron Microscopy (SEM)

Brains (n = 5) were fixed and postfixed as described above, and dehydrated in a graded ethanol series to 100%. The samples were mounted on conductive tabs and vacuum dried in a CPD 030 BAL TEC Critical Point Dryer followed by sputter-coating with gold for 2 min. Images were obtained using the JSM 5310 scanning electron microscope.

### Immunocytochemistry

Dissected brains (n = 8) were immersion-fixed with 4% paraformaldehyde in PB for 12–24 hours. After rinses in PB, the brains were sectioned sagittally with a vibratome (100 µm). Sections were processed using standard immunohistochemical methods, as described previously (e.g., [6]). The following antibodies were utilized: rabbit anti-5-HT (1∶1000; Immunostar, Inc., Hudson, WI), mouse anti-glutamine synthetase (1∶100; Becton Dickinson), goat anti-rabbit IgG Cy2 and goat anti-mouse IgG Cy5 (each at 1∶100; Jackson ImmunoResearch). The mouse anti-glutamine synthetase antibody was raised against sheep glutamine synthetase; the antigen was unavailable for preadsorption testing. Western blots of *P. clarkii* brain homogenate reveal two bands (37 and 45 kDa) in the crayfish brain; 3′Race analysis for GS with three different primers revealed two sequences with about 500 bp difference in size (Genbank Accession #JF738075, JF738076), both of which are highly homologous to GS sequences found in other species (Zhang and Beltz, unpublished results). GS activity in homogenates of the crayfish brain also was confirmed with a glutamyl-transfer assay (Zhang Y and Beltz BS [2010] Soc Neurosci Abstr 36: 737.14). Specificity of the rabbit anti-5-HT antibody, which was generated against synthetic serotonin coupled with bovine serum albumin, was confirmed by preadsorption with the immunogen provided by Immunostar, Inc. Specificity testing done by Immunostar, Inc. has ruled out cross-reactivity of this antibody with 5-hydroxytryptophan, 5-hydroxyindole -3- acetic acid, and dopamine.

Prior to mounting, sections also were treated with the nuclear stain propidium iodide (PI; 10 µg/ml; 10 min at 18–20°C; Sigma). Sections were mounted on slides with Fluoro-Gel, and examined and imaged using a Leica TCS SP5 confocal microscope equipped with argon 488 nm, and 405, 561 and 633 nm diode lasers. Serial optical sections were taken at 1-µm intervals and saved as both three-dimensional stacks and two-dimensional projections.

In some experiments, immunocytochemical techniques were combined with labeling for *Amaranthus caudatus* lectin (n = 10). Lectins are specific carbohydrate-binding proteins that have proven useful for visualization of blood vessels in vertebrate species [16]. Generally, interactions of lectins with the vertebrate endothelium occur through binding to specific glycoprotein moieties on the luminal surface. Because the interaction of lectins with cells is frequently species-specific, comprehensive studies to address selectivity issues are required. However, in this paper we have used *Amaranthus caudatus* lectin (ACL) as a morphological marker only, as in previous studies in *Panulirus argus*, where ACL is described as intensely labeling arterioles in neuropil and cell clusters [10]. In *P. argus*, ACL labeling also distinguished perivascular cells from surrounding neuronal somata. In *P. clarkii*, ACL did not show such selectivity for cell types and was not specific for vasculature structures alone; nevertheless, ACL did label the lumen of blood vessels and made it possible to follow the track of the vascular elements near the niche.

Vibratome sections were incubated in *Amaranthus caudatus* lectin (ACL) conjugated to fluorescein isothiocyanate (Vector, Burlingame, CA) for 4 hours at room temperature. The ACL was diluted 1∶1000 in the secondary antibody for glutamine synthetase (GS), for which the sections were also labeled. Finally, all sections were treated with the nuclear stain DAPI (4′,6-diamidino-2-phenylindole; Molecular Probes). Cover slips were applied as described above.

### Dye Fills of Niche Cells

he morphologies of niche cells were revealed by filling with Lucifer yellow CH dilithium salt (Sigma, #L0259) or Alexa Fluor® 568 hydrazide, sodium salt (Molecular Probes, #A-10441) (n>20). Niche cells were penetrated with sharp glass electrodes and stained by iontophoretic injection with either dye using hyperpolarizing current pulses of up to 10 nA (500 msec duration; 1 Hz frequency) for 10–15 minutes. Following dye injection, preparations were rinsed in crayfish saline, fixed in 4% paraformaldehyde for 12–24 hours, rinsed in PB and mounted in Fluoro-Gel. Dye-filled cells were examined and imaged with the confocal microscope, as described above for immunocytochemical preparations.

## Results

### Features of the Crayfish Neurogenic Niche

**Figure 2:**
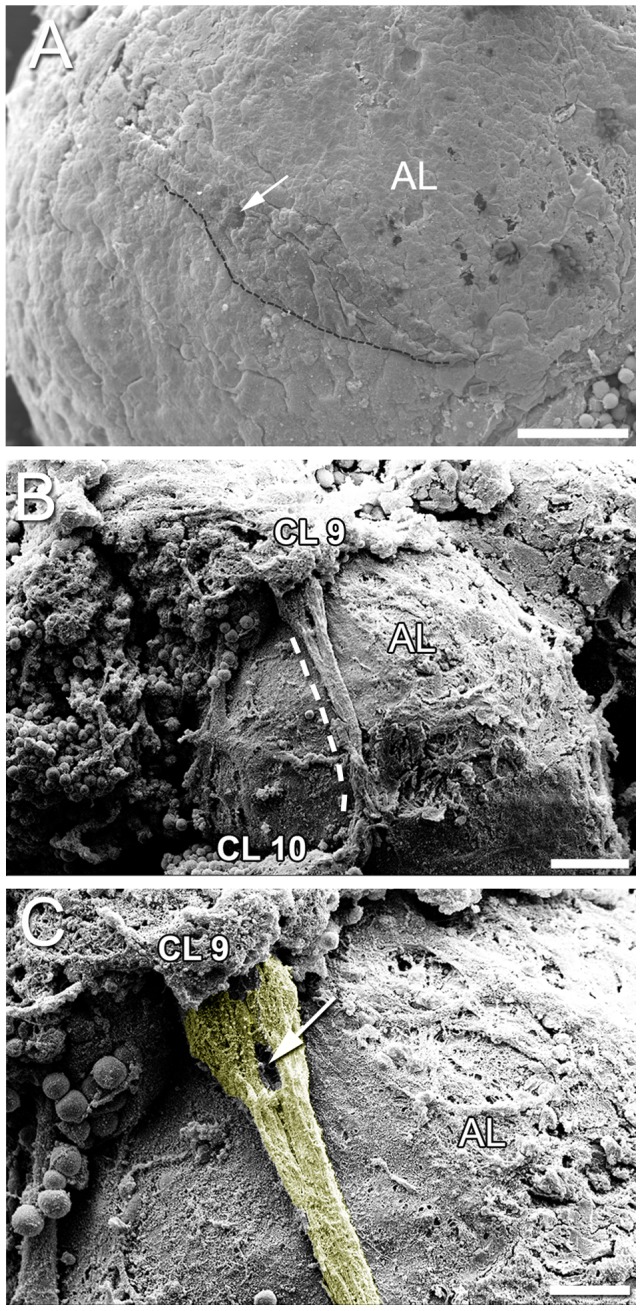
Scanning electron micrographs of the brain showing the niche and migratory streams. (A) The neurogenic niche is seen as a swelling in the central part of the migratory stream on the surface of the accessory lobe (AL). The dotted line delineates the posterior edge of the niche and proximal part of the streams. Note the vascular cavity (arrow) centrally in the niche. (B) The migratory stream on the surface of the accessory lobe (AL) (the dotted line marks the anterior edge of the stream) and the two clusters of cell bodies, cluster 9 (CL 9) and cluster 10 (CL 10). Note that anterior is to the left in the image, and medial is at the top. The niche is not observed, but appears to be obscured by connective tissue. (C) Higher magnification of (B). The migratory stream broadens as it approaches cell cluster 9 (the stream is colorized yellow). The depression (arrow) marks the position of a cell and its processes. Scale bars: (A, B) 50 µm; (C) 20 µm.

The niche appeared by SEM as a bulge roughly mid-way between the cell clusters, filled with swellings that define individual cells and a depression that delineates the vascular cavity (Figure 2 A). The niche and migratory streams were observed on the ventral surface of the brain, attached to the accessory lobe, and extending from the cluster 10 cell bodies (lateral to the accessory lobe) to the medial cluster of cell bodies composing cluster 9 (Figure 2 B). Along the streams, cells were seen as spindle-shaped impressions (Figure 2 B and C). Near the cell clusters, the streams broadened (Figure 2 B and C).

**Figure 3:**
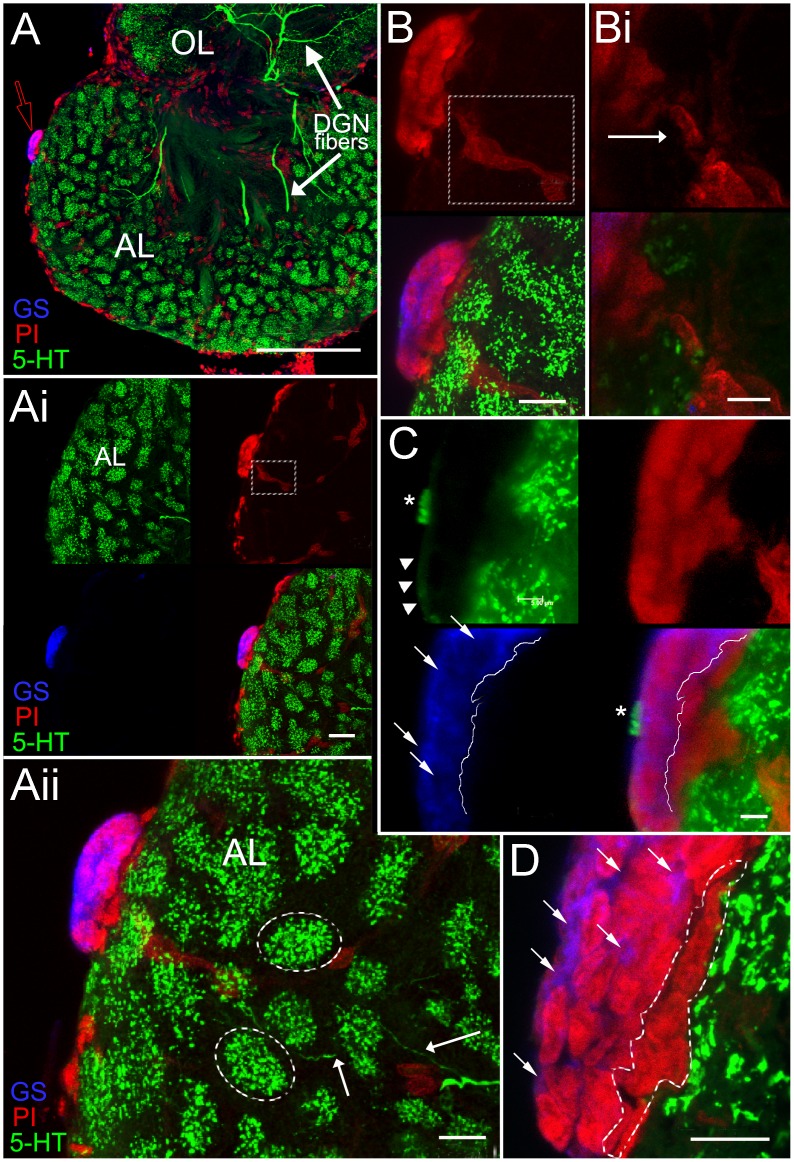
Stacked confocal scans of a 100 µm sagittal section in a *P. clarkii* brain show the neurogenic niche (A–D), stained for GS (blue), protruding from the ventral surface (arrow, red outline) of the accessory lobe (AL). Serotonergic labeling (5-HT) in the olfactory lobe (OL) and AL (A–D, green) reveals fibers from the Dorsal Giant Neuron (DGN) innervating these lobes (A and Aii; white arrows). 5-HT labeling of individual glomeruli (dotted outlines in Aii) and DGN fibers (Aii, arrows) are clearly shown in this section of the AL. Magnifications of this section, including images deeper into the tissue (Ai, B, C) display the three separate laser channels representing the nuclear stain, propidium iodide (PI, red), serotonin (5-HT, green) and glutamine synthetase (GS, blue); PI and GS labeling together reveal the multi-layered aspect of the niche when the brain is sectioned sagittally (B, C, D). In C and D, white arrows indicate the GS-labeled cytoplasm (blue) of the niche cells, demonstrating that the deepest, most dorsal niche layer (outline in D) does not label for GS (dotted line in C, lower panels). The PI labeling cells also delineates the trail of cells, composing a blood vessel that extends from within the AL into the niche itself (Ai, white square; B,white square; Bi, arrow). Higher magnifications show a serotonin immunoreative “crown” of terminals within the outer, ventral layer of the niche (C, asterisk); a weakly immunoreactive serotonergic fiber(s) (arrowheads) is observed connecting to this serrated-like region. Scale bar: (A) 200 µm; (Ai, Aii, B) 20 µm; (D) 10 µm; (Bi, C) 5 µm.

**Figure 4:**
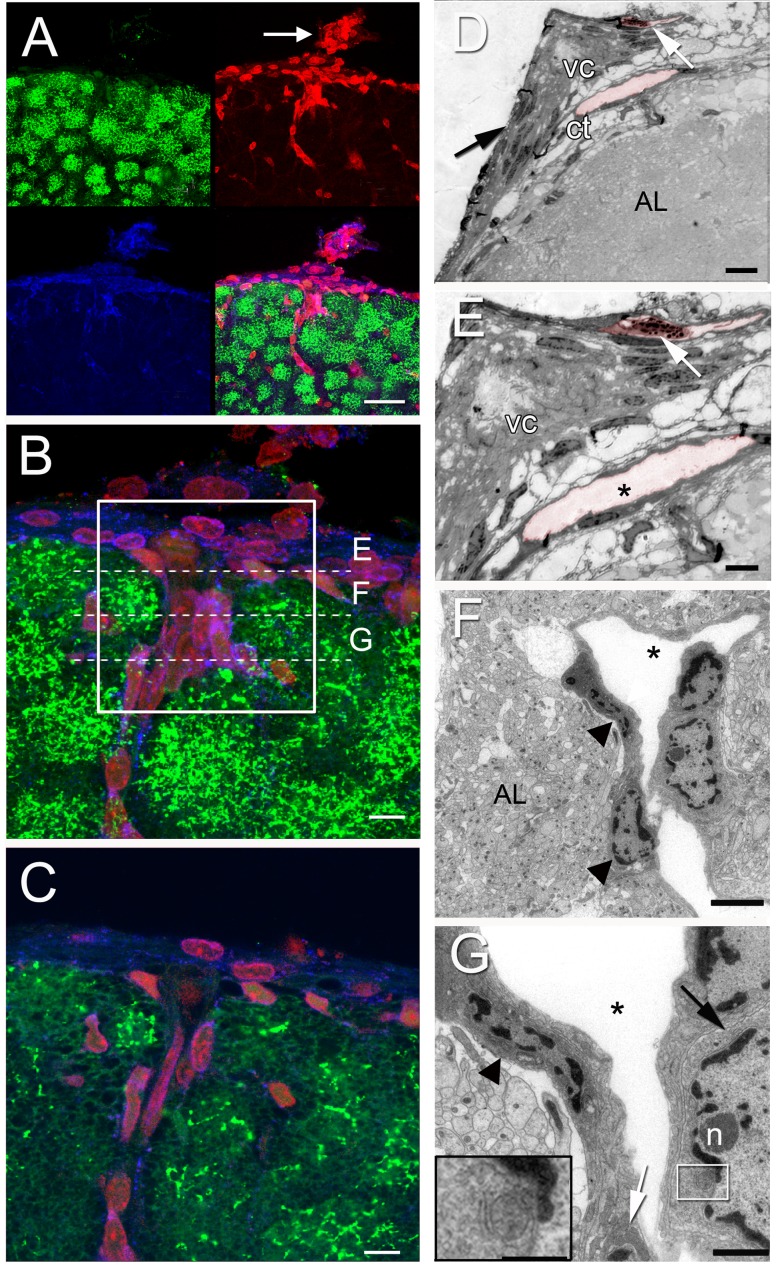
Confocal images of a sagittal niche section (A–C) with separate channels displayed in A (5-HT, green; GS, blue; PI, red; arrow pointing to the niche in A), reveal more dorsal aspects of the niche (B and C) and how cells are organized in regions just below the niche. Corresponding semi-thin (E) and ultra-thin sections (F–G) through regions indicated in B reveal perivascular cells lining a blood vessel. (D, E) A semi-thin section in two magnifications. Note the vascular cavity (vc) and the lumen of two vessels, one below and one above the niche (colorized in pink). Within one of them a granular hemocyte is evident (white arrow). Observe the thin layer of connective tissue limiting the outermost part of the niche (black arrow, D), and in the innermost region, the loose connective tissue (ct) where there is a vessel. (F, G) Electron micrographs of a vessel dorsal to the niche in two magnifications, showing the perivascular cells (arrowheads) lining the vessel, with nuclei composed of heterochromatin apposed to the nuclear envelope, and euchromatin, which constitutes the major part of the nucleoplasm. Also, a nucleolus (n) is seen in one of the nuclei. (G) The cytoplasm of the perivascular cells (arrowhead) showing mitochondrial profiles (white arrow and insert [higher magnification of area limited by the white square]) and many endoplasmic reticulum cisternae (black arrow). Accessory lobe (AL in D and F). Scale bars: (A) 50 µm; (B and C) 6 µm; (D) 35 µm; (E) 20 µm; (F) 5 µm; (G) 2 µm; Insert in (G), 1 µm.

Sagittal sections through brains labeled immunocytochemically for glutamine synthetase and serotonin and stained with the nuclear marker PI showed the niche adhering to the ventral surface of the accessory lobe (Figures 3 and 4). In the 15–20 mm carapace length crayfish used in these studies, the niche was 15–20 µm in depth (dorsal-ventral plane) and 60–80 µm in length (anterior-posterior plane). From prior studies it is known that in the third dimension (lateral-medial plane) the niche is 100–200 µm, gradually tapering at each end into the streams containing the processes of the Type I niche cells. Previous studies have shown, however, that the niche enlarges with growth and aging of the crayfish, and therefore these are not fixed values but rather will change with the size of the animal [5].

Three features of the niche and its relationships with surrounding tissues were revealed by sagittal sections: (1) a distinct layered organization of the cells composing the niche (Figure 3 B–D); (2) a connection with a blood vessel that emanates from more central regions of the accessory lobe (Figures 3 B, Bi, C and 4 A–C); (3) a serotonin immunoreactive “crown” of terminals lying on the most ventral surface of the niche, as well as a weakly immunoreactive superficial fiber (Figure 3 C). Intensely labeled serotonergic fibers and glomeruli were also observed in the accessory lobe beneath the niche (e.g., Figure 3 A, Aii, B); the large serotonin-immunoreactive processes in the accessory lobe come from the Dorsal Giant Neuron [11]. The stratified organization of cells in the niche is particularly clear in Figure 3 D, where flattened, elongate cells compose the most dorsal layer, next to the accessory lobe. The blood vessel emerging from more central regions of the accessory lobe, labeled with PI, forms a continuous column connecting with the most dorsal layer in the niche (Figures 3 B, C and 4 A–C).

**Figure 5:**
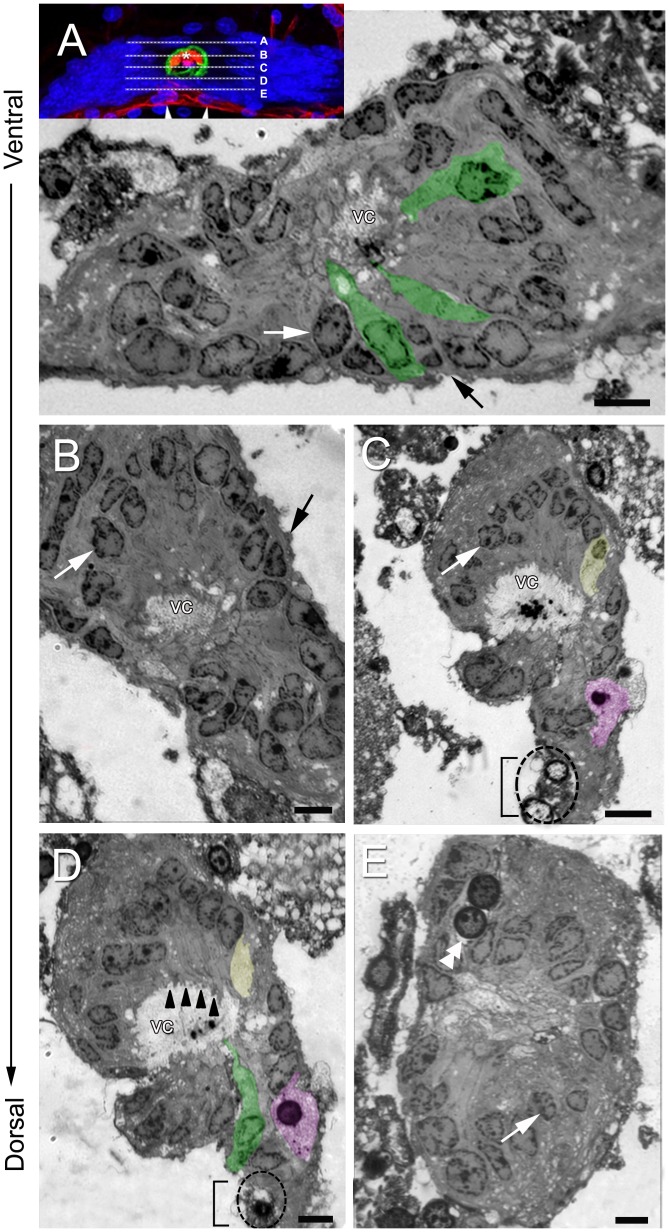
Semithin sections (ventral to dorsal sequence) of the crayfish niche stained with toluidine blue showing four types of cells, the outer edge of the niche and the central vascular cavity (vc). Note that the images displayed in B–E are turned 90° relative to the image displayed in (A). (A, B) Type I cells (white arrows) are the most common, and have strongly stained cytoplasm and nuclei that are, in general, cuboidal or elongate. The cytoplasm of these cells lines the vascular cavity (examples are colorized green) and has fine extensions projecting into the lumen. The outer edge of the niche is covered with a thin layer of connective tissue (black arrow). The insert in (A) shows the position of corresponding semithin sections through the niche (A–E). (C, D) In more dorsal positions, in addition to Type I cells and their projections to the lumen (irregular cytoplasmic process – arrowheads in D), a Type II cell (colorized yellow) and Type III cells (one example is colorized purple) are identified. Type II cells have a nucleus with euchromatin and scarce heterochromatin, and a clear cytoplasm. The image in (D), which is 1.0 µm more dorsal than that in (C), shows only the cytoplasm of the Type II cell). Note two round cells (dotted circle) near to the emergence of the streams (bracket) with features that are distinct from the other cell types. These resemble Type IV cells (see below). Type III cells appear as amoeboid-shaped cells. Two type III cell nuclei are seen in the image (C), however only one appears in the image (D). Note the clear cytoplasm displayed by type III cells. In the center of the vascular cavity, there is intensely-stained material with a globular organization. The stream emerging from the niche is identified by the bracket. (E) Type IV cells (double arrowhead) have a spherical profile and are distant from the vascular cavity, near the emergence of the streams. This cell type frequently appears with paired nuclei and a clear cytoplasm. Type I cells are also seen (white arrow) in this image. Scale bars: (A–E) 12 µm.

**Figure 6:**
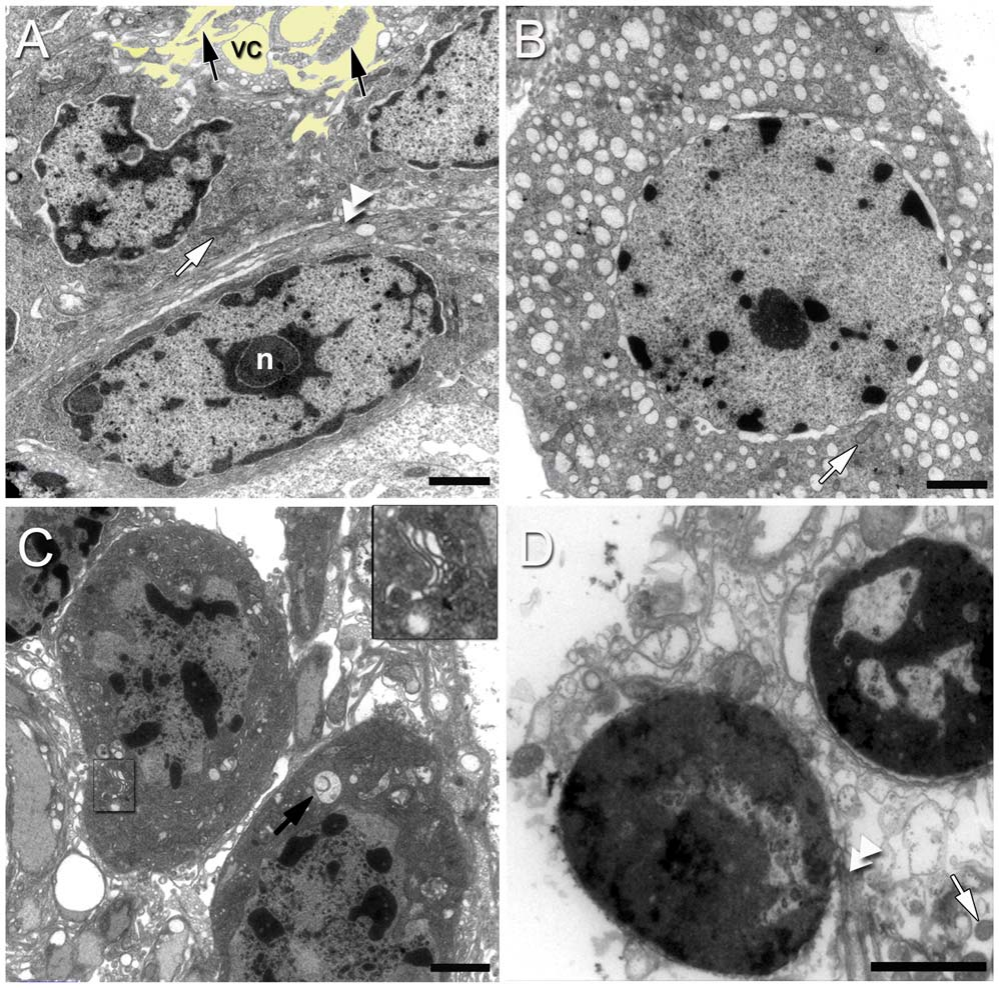
Ultrastructure of the neurogenic niche cells. (A) Type I cells have nuclei that are frequently elongated; these contain both heterochromatin and euchromatin, and a nucleolus (n) is often seen. Mitochondrial profiles (white arrow) and rough endoplasmic reticulum (double arrowhead) are abundant. In the upper part of the image note the processes (arrows) projecting into the vascular cavity (vc).The vascular cavity is colorized yellow to highlight the processes of the niche cells projecting into this area. (B) Type II cells have a nucleus containing abundant euchromatin and sparse heterochromatin. Note mitochondrial profiles (arrow) and many vesicles in the cytoplasm. (C) Type III cells display a nucleus composed of clumps of heterochromatin. Their cytoplasm contains vesicles, some of them with membranes in their interior (black arrow), as well as a Golgi apparatus (outlined in C and magnified in the insert). (D) Type IV cells are found in pairs, suggestive of cell division, however, no shared plasma membrane between the two nuclei is visible. In the cytoplasm observe mitochondrial profiles (white arrow), dilated vesicles and cisternae of the endoplasmic reticulum (double arrowhead). Scale bars: (A–D) 2 µm. Insert in (C), 0.5 µm.

**Figure 7:**
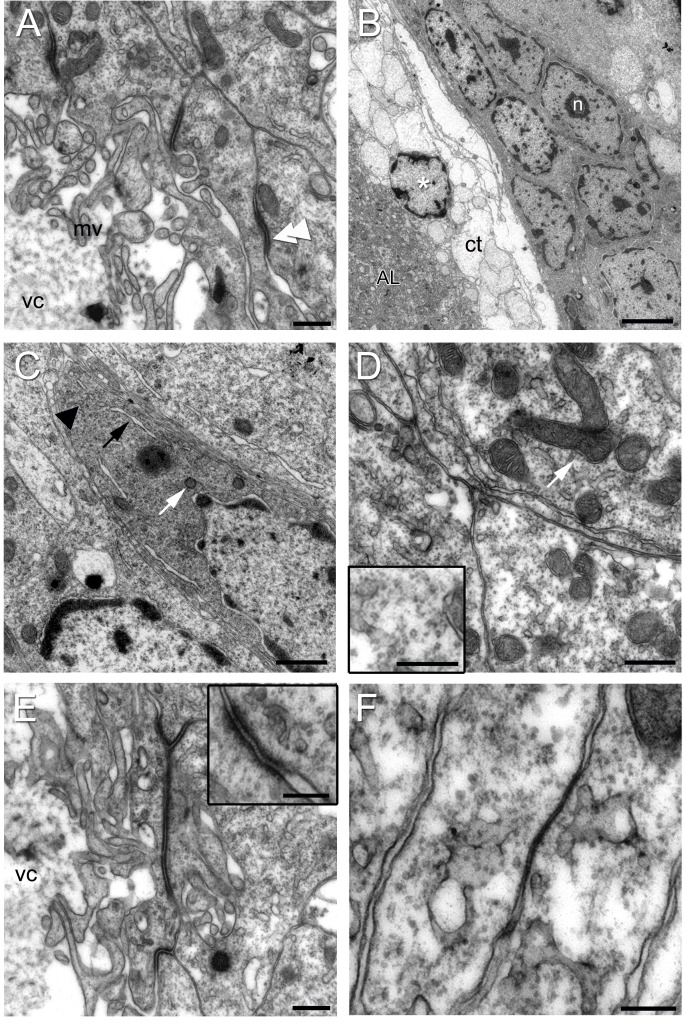
Electronmicrographs of Type I cells. (A) Region of the niche near the vascular cavity (vc) showing Type I cells lining the edge of the cavity. Note the microvilli (mv) projecting into the cavity. *Zonulae adherens* join adjacent cells (double arrow head). (B) Type I niche cells near the emergence of the streams have elongated nuclei containing heterochromatin and euchromatin. A nucleolus (n) is apparent. Connective tissue (ct) is adjacent to the dorsal surface of the niche facing the accessory lobe (AL); a cell with features of a crustacean hemocyte (asterisk; hyaline cell, e.g., [56], [57]) is located within this tissue, although a blood vessel is not apparent. (C) Type I cells contain an abundance of mitochondrial profiles (white arrow), rough endoplasmic reticulum (black arrow), polysomes (arrowhead) and vesicles. (D) Cytoplasm of type I cells in high magnification: Observe the numerous mitochondria (arrow) and microtubules, seen in detail in the insert, lower left. (E) Another view of the apical surface of a Type I cell, showing a *zonula adherens* between adjacent cells, as well as microvilli projecting into the lumen of the vascular cavity (vc). The insert (upper right) shows a higher magnification of a *zonula adherens*. (F) Type I cells united by a septate junction. Scale bars: (A) 0.5 µm; (B) 5 µm; (C) 1 µm; (D, E) 0.5 µm; Inserts in (D) 0.1 µm and (E) 0.5 µm; (F) 0.2 µm.

**Figure 8:**
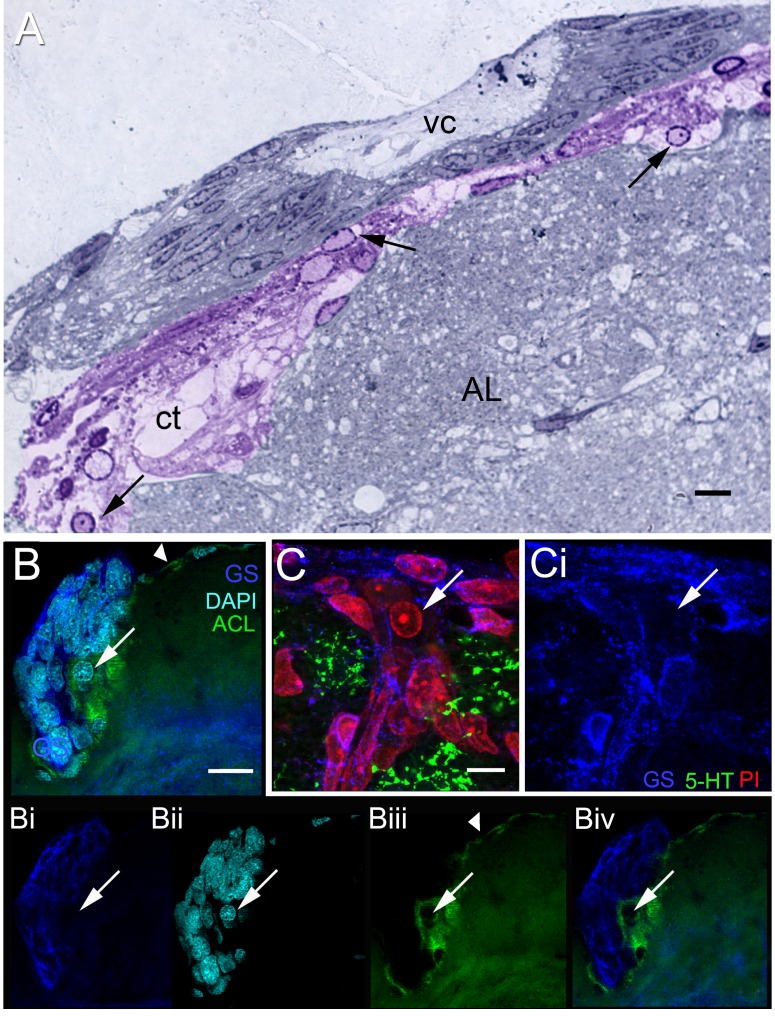
The niche and surrounding tissues. (A) Semi-thin section showing the niche and the central vascular cavity (vc). The connective tissue (ct) below the niche (colorized purple) has many cells (arrows) similar to that seen in Figure 7 B (asterisk), with features resembling hemocytes. (B) A stacked confocal image of the neurogenic niche labeled with glutamine synthetase (GS, blue) and stained for DAPI (cyan) and the lectin ACL (green); separated emission channels are shown in Bi (GS), Bii (DAPI), Biii (ACL). ACL delineates the vasculature lying underneath (as in A) and enveloping the niche (compare also with Figure 4 D, E). Biv is the composite of the GS and ACL channels only, showing the close apposition of the two labels, but no overlap. A cell (arrow in B) without cytoplasmic GS labeling and with the morphological features of hemocytes is detected in the vasculature/connective tissue (ACL). (C) A confocal image (serotonin, green; GS, blue; PI, red) reveals a cell (arrow) with the morphological characteristics of a hemocyte (as in 8A, B) in the most dorsal layer of the niche, which also (as in 8B) is not delineated by GS labeling (Ci, single channel for GS). Cells lining the vasculature (perivascular cells) do contain GS label. Scale bar: (A) 10 µm; (B) 20 µm; (C) 10 µm.

In vivo, the entire brain including the niche is enveloped by the brain sheath, or perineurium, which was removed after fixation in the studies presented here. Semi-thin sections showed that the niche itself is covered on its ventral surface by a thin layer of connective tissue (Figure 5 A,B), within which blood vessels are seen (Figure 4 D, E). In the innermost (dorsal) region facing the accessory lobe, the niche is bounded by several layers of loose connective tissue, within which differently sized blood vessels are located (Figure 4 D–E). These images therefore suggest that the niche is enveloped by vascular elements, with the exception of the most ventral region where the niche would be bathed by interstitial fluids. Portions of blood vessels that run below the vascular cavity were lined by perivascular cells (terminology of Abbott [17]), which share many similarities with the Type I niche cells (compare Figure 4 F, G with Figure 6 A; Figure 7). These ultrastructural observations revealed that cell types in the niche and those associated with the vasculature have nuclei containing clumps of heterochromatin apposed to the nuclear envelope, as well as euchromatin that constitutes the major part of the nucleoplasm; a prominent nucleolus was often present. The cytoplasm of the perivascular cells contained mitochondrial profiles and a basement membrane in the adluminal region (Figure 4 G, inset), which is a feature that occurs in the vessels of other invertebrates such as the ascidians [18]. Hemocytes were also encountered within the blood vessels surrounding the niche (Figure 4 D, E) and within the connective tissues lining the dorsal surface of the niche (Figure 8 A, C).

### Characteristics of Cell Types Composing the Niche

Semi-thin sections examined by light microscopy showed that the niche is constituted by three types of cells first observed by Zhang et al. [5] (Figures 5 A–D and 6 A–C), plus an additional fourth cell type (Figures 5 E and 6 D). Type I cells were found at all levels in the niche and were by far the most numerous. The other three cell types (Figures 5 C, D and 6 B, C), described in more detail below, were only seen in the more dorsal regions of the niche, generally close to the vascular cavity which is enlarged and particularly distinct in central regions of the niche. This positioning of different cell types parallels the stratified organization of the niche observed in the labeled sagittal sections viewed with confocal microscopy (Figure 3 B–D).

**Figure 9:**
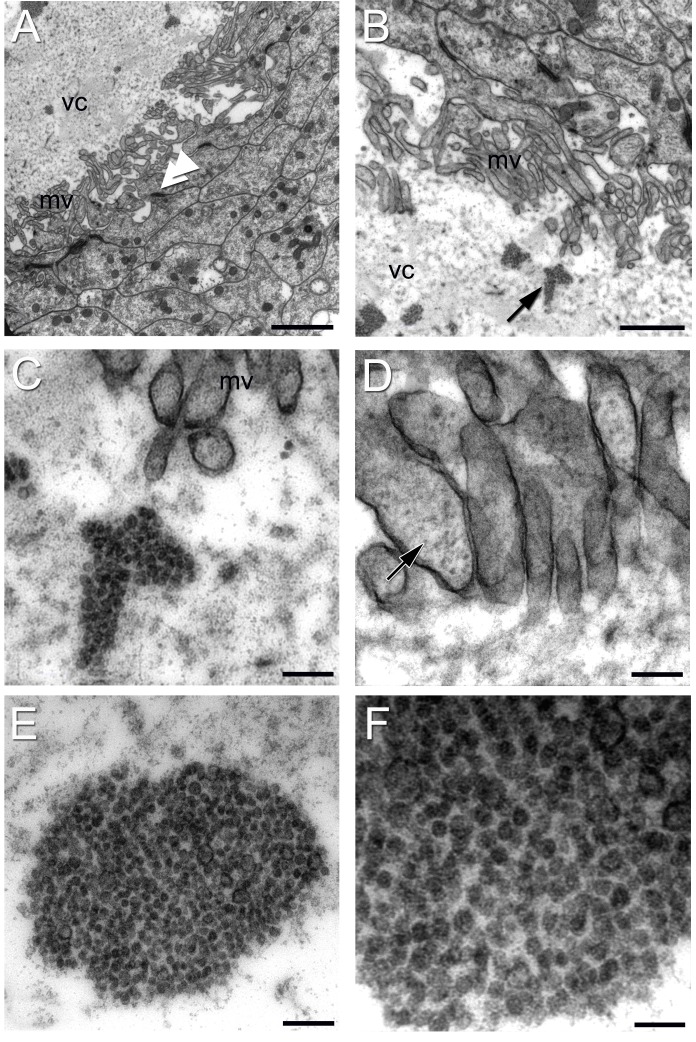
Electron micrographs of the vascular cavity. (A) Type I cells lining the vascular cavity (vc), which contains granular electron dense material. Note the microvilli (mv) projecting to the lumen of the cavity. Also observe the *zonulae adherens* joining adjacent cells (double arrow head). (B) A higher magnification showing the rim of the vascular cavity in another area. Note again the electron dense material with a globular organization, sometimes aggregated in clumps (black arrow) in the lumen of the vascular cavity. Clumps are better seen in (C). (D) High magnification of microvilli of a type I cell containing many microfilaments (arrow). (E, F) Higher magnifications of the round blobs composing these clumps. Scale bars: (A) 2 µm; (B) 1 µm; (C) 0.2 µm; (D, E) 0.2 µm; (F) 0.1 µm.

**Figure 10:**
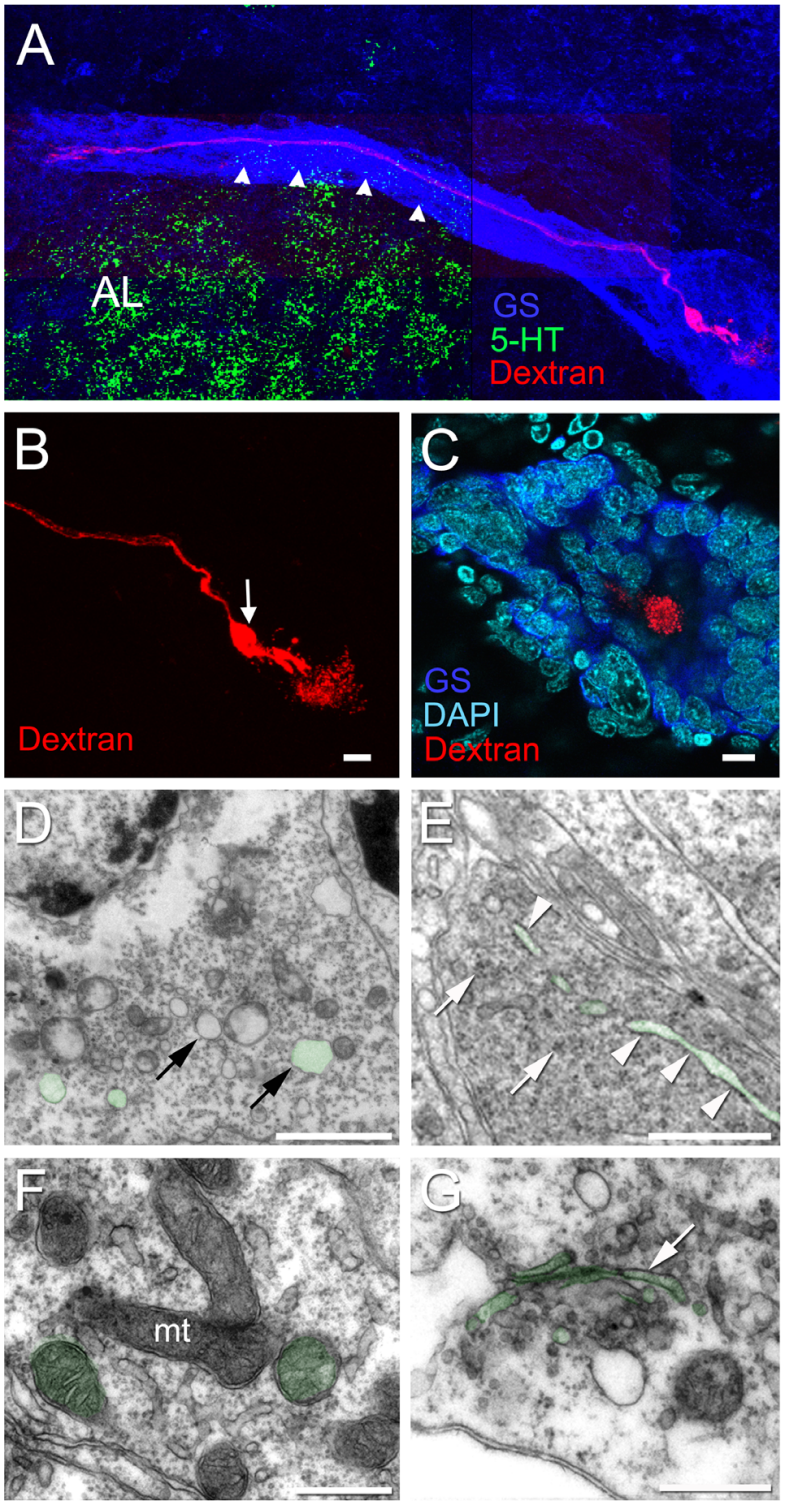
Alex Fluor® 568 hydrazide dye injection of Type I niche cells reveals their bipolar morphology. Each Type I cell has a long process that stretches to either cluster 10 or cluster 9. (A) Montage showing the long process of a dye-injected cell, projecting towards cluster 10. Arrowheads point to the fascicle of Type I cell processes, labeled for GS, that form the migratory stream, and a short process that extends to the vascular cavity (B). Arrow in (B) points to the cell body, where the dye was injected. The injection of dye into Type I cells results in deposition of the dye in the vascular cavity (B and C). (C) A dye fill of a different Type I cell in which the niche is labeled for GS and stained with DAPI, shows the vascular cavity containing Alexa Fluor®, surrounded by the niche cells. (D–G) Additional ultrastructural details of the cytoplasm of Type I cells. (D) Vesicles (arrows). (E) Note rough endoplasmic reticulum (arrowheads) and some clumps of ribosomes (arrows). (F) Mitochondria (mt). (G) Golgi apparatus (arrow). Examples are colorized green. Scale bar: (B, C) 10 µm; (D) 2 µm; (E) 1 µm; (F) 0.5 µm. (G) 0.5 µm.

Type I cells, which were observed with both light and electron microscopy (Figures 5, 6 A, 7, 8 A and 9 A,B), are by far the most abundant cells in the niche. Prior studies have shown that Type I cells are bipolar, with short cytoplasmic processes projecting to the vascular cavity and longer processes, which constitute the migratory streams, to cell clusters 9 and 10 (Figure 1 B, C) [4]. Type I niche cells contain large, cuboidal or elongated nuclei (Figure 5) composed of both heterochromatin and euchromatin, and a nucleolus was often seen (Figures 5, 6 A, 7 B and 8 A). Within the cytoplasm there are mitochondrial profiles (Figures 7 A, C, D and 10 F), rough endoplasmic reticulum (Figures 6 A, 7 C and 10 E) polysomes (Figures 7 C and 10 E), vesicles (Figures 6 A and 10 D) and Golgi apparatus (Figure 10 G). The preponderance of ribosomes explains the basophilic aspect of this cell type when seen by light microscopy. Cytoskeletal elements, such as microtubules (Insert of Figure 7 D) also were observed.

The surfaces of Type I cells facing the vascular cavity displayed uneven borders consisting of thin and irregular cytoplasmic processes seen both by light (Figure 5) and electron (Figures 6 A, 7 A, E and 9 A, B) microscopy. The presence of microfilaments (Figure 9 D, insert) is typical of microvilli. In some areas this boundary resembles a brush border, indicating molecular transport and uptake functions. A possible secretory role for these cells also is suggested (see details below, in *The vascular cavity*). Cilia were not observed associated with the Type I cells.

Another striking feature of the Type I cells lining the vascular cavity were the junctional complexes consisting of *zonulae adherens* (Figures 7 A, E and 9 A, B) and septate junctions (Figure 7 F). Septate junctions are the primary occluding junction in invertebrates, and are thought to be functionally equivalent to vertebrate tight junctions [19], [20], [21], [22]. Gap junctions were not observed between these cells.

Type II cells (Figures 5 C and 6 B) contain a nucleus with euchromatin and scarce heterochromatin, and a clear cytoplasm. The euchromatin and heterochromatin can be seen in detail with electron microscopy (Figure 6 B). In the cytoplasm, in addition to mitochondrial profiles, a large number of vesicles was observed (Figure 6 B), which is probably why these cells were poorly stained for light microscopy (Figure 5 C).

Type III cells may be characterized by light microscopy as an ameboid-shaped cell (Figure 5 C, D). The EM revealed a cell type with a nucleus composed of clumps of heterochromatin and a cytoplasm containing vesicles, some of which had membranes in their interior (Figure 6 C). Endoplasmic reticulum and Golgi apparatus (Figure 6 C and insert) were also observed.

The Type IV cells observed in this study were spherical and located away from the vascular cavity, near the emergence of the migratory streams formed by the long processes of the Type I cells (Figures 5 E and 6 D). These cells appear to have just divided, based on their proximity to each other, similar size and appearance, and their location. In previous studies we have noted that mitotic 1^st^-generation precursors are often seen near the migratory streams [5]. The cytoplasm of type IV cells displays many mitochondria, dilated vesicles and cisternae of endoplasmic reticulum (Figure 6 D).

### The Vascular Cavity

**Figure 11:**
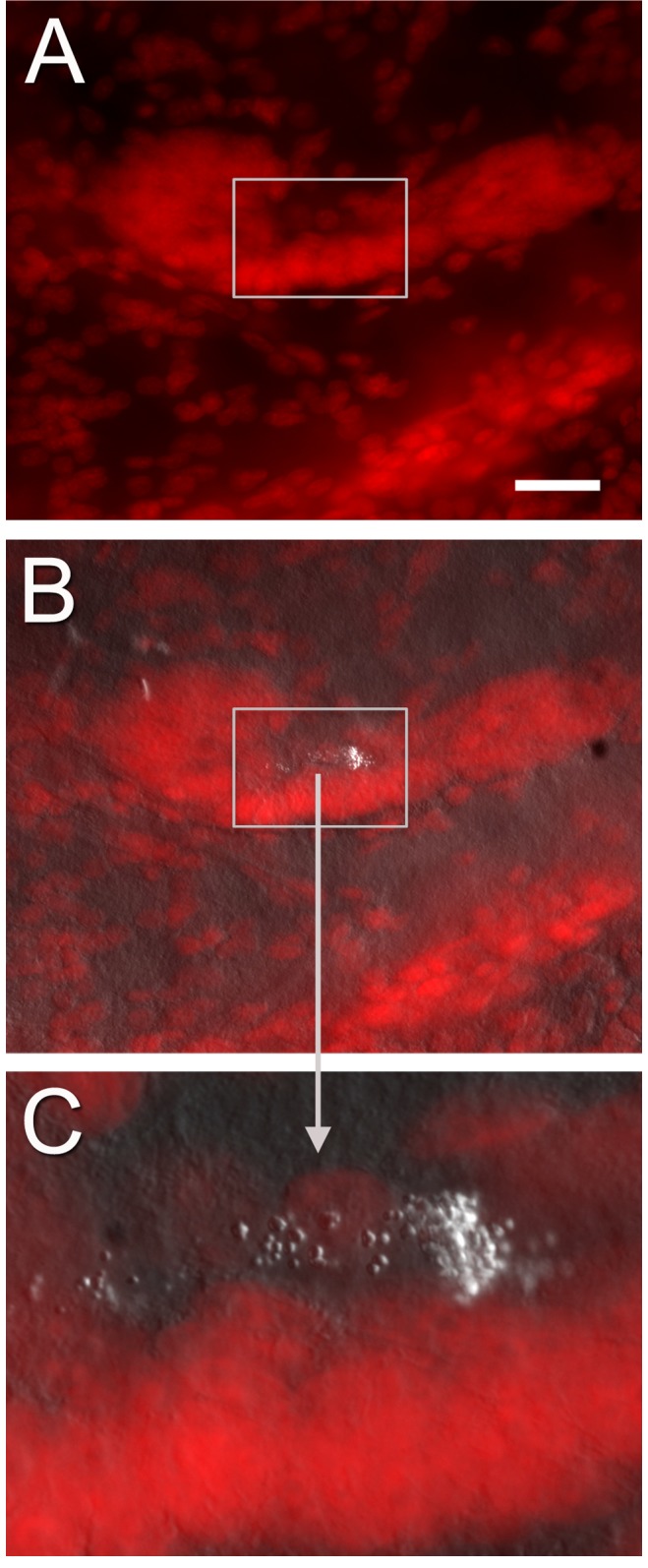
Combined confocal images (B, C) of PI-labeled cells in the niche (A) and Nomarski optics showing bubble-like material in the vascular cavity (higher magnification in C; rectangle in A and B). These structures of unknown origin are also observed in EM sections (e.g., 9B–C, E–F) that show the material to be composed of electron dense particles. Scale bar: (A) 40 µm.

Within the lumen of the vascular cavity, located centrally in the crayfish niche, electron dense material with a globular organization was observed by light microscopy (Figure 5 C, D). At the ultrastructural level, this material had a granular appearance and was found in clumps dispersed irregularly within the lumen (Figure 9 A–C, E, F). The round blobs composing these clumps have fairly homogeneous diameter (30–35 nm). These clumps appeared as “bubble-like” material when viewed with confocal and Nomarski optics (Figure 11).

The microvillar border of the niche Type I cells indicates a selective transport function for these cells. In addition, the preponderance of Golgi apparatus (Figure 10 G), vesicles (Figure 10 D), rough endoplasmic reticulum (Figure 10 E) and mitochondria (Figure 10 F) may suggest that the Type I niche cells are involved in a secretory function. In support of this possibility, iontophoretic injections of Type I niche cells with Lucifer yellow or Alexa Fluor® 568 hydrazide invariably resulted in leakage of the dye into the vascular cavity (Figure 10 A, B).

## Discussion

The results presented here document several notable features of the neurogenic niche in the crayfish brain: (1) The niche is a layered structure, and different cell types are present in the ventral vs. dorsal layers. (2) Blood vessels are embedded in the connective tissues that surround the niche, and vessels from accessory lobe tissues on the dorsal side of the niche contact and appear to communicate with the niche. (3) Three cell types previously reported [5] were confirmed and characterized in the present study, and a fourth cell type was identified. (4) The Type I cells form a microvillar border at the vascular cavity, and zonulae adherens and septate junctions are present at this boundary, suggesting a selective transport function for these cells. (5) The leakage of iontophoretically injected dyes into the vascular cavity and the presence of organelles consistent with a secretory function, also characterize the Type I niche cells. (6) Granular material is found in the vascular cavity, although the nature of this material and its significance are not known. (7) Serotonin-immunoreactive terminals form a “crown” of tissue on the ventral surface of the niche. The cellular source of these terminals has not yet been identified. These features are discussed below.

### Cellular Layering of the Niche

The stratified nature of the niche was not previously recognized. In the current studies, the sagittal sections clearly reveal this cellular layering, a feature that was confirmed with semi-thin and thin sections, which showed the presence of different cell types in the deeper, dorsal regions compared with more ventral areas. The most dorsal cell layer is contiguous with connective tissue containing blood vessels.

### Relationship between the Vasculature and the Niche

A close relationship with the vasculature had been noted in earlier studies showing that the niche sits on top of a blood vessel (Figure 1D [4]). In addition, injecting fluorescently-labeled dextran into the dorsal artery supplying the *P. clarkii* brain [4] or into the pericardial sinus [6] results in the presence of labeled dextran in the vascular cavity. We have found that both dye concentration and the time between dextran injection and sacrifice are critical for visualizing the label. We therefore assume that the failure by Song et al. [23] to replicate these dye-fills of the vascular cavity in *P. clarkii* relates to technical differences, although details of their dye-injection protocol were not provided. Some type of physical connection between the cavity and the vasculature also is suggested by the fact that dye injected into the cavity itself invades nearby vessels (Sandeman and Beltz, unpublished results).

While communication between the niche and the circulation has been clearly demonstrated, the source of the blood vessel servicing the niche and the anatomical connections between the niche and the vasculature have not been visualized previously. The current studies illustrating the highly vascularized region contiguous with the niche confirm a close relationship between the niche and the underlying vasculature. Images show that the niche in the mature brain is nearly encompassed by blood vessels that are embedded in connective tissues, suggesting a retia-like complex of fine channels. These findings also extend developmental studies showing that the crayfish niche develops in concert with angiogenesis around the time of hatching [24]. Angiogenesis also has been associated with neurogenesis in vertebrate systems [25], [26].

The vascular system has been receiving increasing attention as a common feature of all stem cell niches, including those in the nervous system [9], [26]. Indeed, dividing neuronal precursors in vertebrates are found in dense clusters associated with the vasculature, and roughly 37% of all dividing cells are immunoreactive for endothelial markers [27], [28]. Even in pathological conditions, the injured brain can release a variety of diffusible mitogens, such as vascular endothelial growth factor (VEGF) which has been implicated in enhancement of proliferation of progenitor cells [29], [30]. An extensive list of neurovascular interactions that influence neural stem cells has been compiled, establishing that the function of neurogenic niches in vertebrates is dependent on a close relationship with the vasculature [26].

### Niche Cell Types and Proposed Functions

Our data demonstrate four distinct cell types in the crustacean neurogenic niche. Type I cells are by far the most numerous. These cells line the vascular cavity and have specializations that provide clues to their functions. It is not known whether the other cell types are related to the Type I cells, for instance, a developmental progression of a single cell type, or if these play distinct roles. The roles of Type II and Type III cells are unknown, although the ameboid shape of Type III cells may suggest that these cells are not resident niche cells but are transitioning to the niche. The position, shape and size of Type IV cells is consistent with an identity as daughters of the 1^st^-generation niche precursor cells, the 2^nd^ generation cells that will contact the processes of the Type I cells and travel along the migratory streams.

As prior studies have demonstrated communication between the vascular cavity and the circulatory system, the rim of the vascular cavity serves as the interface between the niche cells and the hemolymph, Type I cells form a microvillar border on the edge of the vascular cavity. These microvilli increase membrane surface area and are an important factor in transport physiology, as these provide enormous potential for solute exchange. The lack of cilia suggests that the flow across the microvillar border is passive, resulting from hemolymph movement in the vasculature rather than from active forces generated by niche cells. These cells also have organelles consistent with a secretory function, and dye injections of the bipolar Type I cells show that dyes leak into the cavity, supporting this possibility.

Type I cells also are connected to each other via junctional complexes composed of *zonulae adherens* and septate junctions. Septate junctions of the Type I cells, as in other invertebrate cell types, are characterized by a ladder-like appearance, and are the occluding junctions that provide the functional equivalent of vertebrate tight junctions to maintain the blood-brain barrier [31], [32]. Septate and tight junctions also share molecular components (e.g., claudins, [33]). Septate junctions usually constitute a belt at the apical region of epithelial cells, as they do in the Type I cells, and are often associated with other types of junctions, such as *zonulae adherens* [34]. Strong immunoctyochemical labeling for cadherin has been observed in a lattice-like structure surrounding the vascular cavity [4]. This lattice is located in the same region where *zonulae adherens* were identified as bands encircling the Type I cells. *Adherens* junctions generally link on the cytoplasmic face to the actin cytoskeleton and contain the transmembrane glycoprotein E-cadherin [35].

It has been proposed that the *zonulae adherens* provide the structural support required to maintain septate (or tight) junctions [36], while the septate junctions maintain a barrier to paracellular flow. Tight junctions consistently occur where highly occluding permeability barriers are required. It is interesting to note, however, that not all tight junctions form a rigid permeability barrier, they can be either tight or leaky [37], [38], and that these properties are dynamically regulated [39]. In addition, there are signals transmitted from tight junctions to the cell interior which can modulate gene expression, cell proliferation and differentiation [40]. Based on the microvillar border and the presence of junctional complexes, we suggest that one function of the Type I cells is to regulate the microenvironment in the niche by controlling the flow of solutes both in and out of the niche, establishing a microenvironment that includes factors that are important for the differentiation of the niche cells. In addition, the large number of mitochondria in the cytoplasm of the cells around the vascular cavity, as well as the frequency of vesicles, may suggest that these cells may participate in a secretory function, since mitochondria are responsible for the high respiratory metabolism of cells and provide the energy necessary for the active transport that the cells undertake during the secretion process.

The microvillar border and junctional complexes found in the Type I niche cells at the border of the vascular cavity are reminiscent of the specializations found in vertebrates at sites where the blood or cerebrospinal fluid interfaces the nervous system [39], [41]. For example, these features are characteristic of the endothelial cells in the walls of capillaries in the brain composing the blood-brain barrier, the epithelial cells of the choroid plexus and the arachnoid cells enclosing the central nervous system. In all of these cases, the epithelial layer has tight junctions between the cells on the apical surface facing the blood vessel, ventricle or cerebrospinal fluid (CSF) space, respectively. These tight junctions provide a selective barrier between the nervous system and blood or CSF, acting as a barrier to flow between cells. In addition, the brush border of microvilli on these epithelial layers increases the surface area for communication [42]. Perhaps not surprisingly, these features also are found in the ependymal cells that form the platform of the subventricular zone generating adult-born neurons in the mammalian brain [43], [44]. There are therefore many parallels between the vascular interface in the crustacean niche, the ependymal layer of mammalian neurogenic regions and areas that form interfaces between the vertebrate CNS and blood-CSF, perhaps suggesting common transport functions of these boundary layers.

### The Vascular Cavity

The nature of the material within the lumen of the vascular cavity in the crayfish niche is not known. Bazin [45] discovered structures in many crustacean species which he named “deutocerebral organs”, but whose function was not known. It has since become clear that the deutocerebral organ is the neurogenic niche described by Sullivan et al. [4], see also [46]. Bazin explored the nature of the luminal substance, a material morphologically similar to that we observed in the lumen of the vascular cavity of *P.clarkii*, by labeling this material with histochemical methods for glycosaminoglycans (alcian blue) and for glycoproteins (periodic acid-Schiff). Based on these studies, we may assume that part of the luminal substance is glycidic. Cells specialized in the synthesis and secretion of glycoproteins that are released into the CSF are located in the subcommissural organ (SCO) of developing chickens, an ependymal differentiation located in the roof plate of the diencephalon [47], [48]. It has been proposed that the SCO secretion participates in ontogenetic processes in the CNS, such as neuronal differentiation, neuronal aggregation and axonal pathfinding [49], [50]. It is possible that the aggregated material found in the interior of the cavity in the neurogenic niche may be released by the Type I cells as signaling molecules that somehow regulate neurogenesis. The fact that Lucifer yellow or fluorescently-labeled dextran injected into niche cells invariably is found labeling the substance in the lumen of the vascular cavity supports the suggestion that the glycidic material may originate in the niche cells.

### The Serotonergic Crown

Serotonin is a powerful regulator of adult neurogenesis in crustaceans [6], [11], [51], [52], [53], and is important in the attraction of hemocytes to the niche in vitro [6]. The presence of serotonin immunoreactivity in the niche is therefore of particular interest.

The serotonergic endings sitting atop the niche were observed previously in wholemounts of the niche [6]. It was suggested that these might be terminals of the Type I niche cells, which project to the vascular cavity and terminate in small boutons [4]. However, the niche cells are devoid of serotonin labeling, an unusual situation if these cells are the source of the serotonin. Another possibility suggested by the current experiments is that the deutocerebral Dorsal Giant Neuron (DGN), which has extensive projections terminating in every glomerulus in the accessory lobe, may project to the niche. While a direct connection between the crown and the DGN has not been demonstrated, fine fibers immunoreactive for serotonin can been visualized superficially in the accessory lobe, in the vicinity of the niche. This is an interesting hypothesis that is being further explored, because the DGN is known to directly regulate the incorporation of BrdU/production of new neurons in the adult crayfish brain [11]. A direct connection between the DGN and niche would therefore be consistent with serotonergic control mechanisms that have already been proposed.

### Comparison between the Niche in P. clarkii and other Decapods Crustaceans

An ultrastructural study of a neurogenic niche in adult lobsters, *Panulirus argus*, has been published [10]. The system producing adult-born neurons in *P. argus* is organized differently with separate niches located within cell clusters 9 and 10; this is in contrast to the single niche found roughly midway between clusters 9 and 10 in crayfish, which supplies neuronal precursors to both cell clusters. At the microscopic level, there are both similarities and differences between the two species. While the niches in *P. argus* are located near vascular elements, no direct communication between the niche and the circulation has been demonstrated. This indicates a different relationship with the vasculature than the direct communication suggested in crayfish (see *Relationship between the vasculature and niche,* above). Several lines of data described above support a more intimate association between the vascular system and the neurogenic niche in *P. clarkii*, than the relationship proposed in *P. argus*.

In the *P. argus* niche, two cell types were identified: “clump-forming cells” (CFCs), which appear to be comparable to Type I cells in *P. clarkii*; and a single αNB (alpha-neuroblast). The cell body of the αNB is positioned in a duct connecting the niche to Cluster 10, while the cytoplasm is found within the central niche cavity; an hourglass-like shape is proposed for this cell, which is presumed to continuously supply the neuronal cell clusters with precursor cells via self-renewing divisions. The same model also been suggested for *P. clarkii*, with “a particularly large proliferating cell that has an invariant location” [10], [23] responsible for adult neurogenesis. Schmidt and Derby [10] therefore propose “based on their large size and privileged location, these cells were identified as putative adult neuroblasts (aNBs). Thus, adult neurogenesis in the olfactory deutocerebrum of decapod crustaceans appears to be maintained by NSCs and neural progenitor cells that are equivalent to those fueling embryonic neurogenesis, NBs and GMCs, respectively.”

In our experiments in *P. clarkii*, a large cell in an “invariant position” has never been observed in adult niches [4], [5], [6], [54], [55] or associated with the protoniche as it emerges [24]. Further, the relatively slow cycling of the cells that constitute the emerging protoniche [24] and the adult niche [6] is in contrast to the rapid cycling of neuroblasts. The anatomical data of Song et al. ([23], also cited in [10]) do show the presence of large BrdU-labeled cells in the *P. clarkii* niche, which we have also observed but not in a fixed location. Further, our studies have shown that the 1st-generation neuronal precursors residing in the niche have geometrically symmetrical divisions [5] and that dividing (BrdU-labeled) cells in the niche do *not* self-renew [6], characteristics that are inconsistent with a neuroblast identity. We have therefore concluded that the niche is not a closed system, and that the niche precursor cell population must be replenished from an extrinsic source [6]. In vitro experiments suggest that a likely source of neuronal precursors in the niche is the vascular system [6].

At the ultrastructural level, there are additional differences between data reported in *P. argus* [10] and our current findings in *P. clarkii*. In *P. argus*, “occasional desmosome-like membrane specializations are present between adjacent processes of different CFCs”. In contrast, in *P. clarkii* we have demonstrated septate and adherens junctions between adjacent Type I cells around the entire circumference of the vascular cavity, suggesting a structural barrier to movement between cells. Further, the elaborate microvillar border on Type I cells at the interface with the vascular cavity indicates a selective transport function for these cells. No microvillar border has been reported for CFCs lining the cavity in *P. argus*.

These contrasting organizational and ultrastructural findings in *P. argus* and *P. clarkii* suggest that there are important differences in the cellular machinery underlying adult neurogenesis in these two species, and which may relate to functional mechanisms. We therefore suggest that data from a single species should not be generalized to all decapod crustacean species.

### Conclusions

Prior studies in the crayfish *P. clarkii* have lead to the idea that cells emerging from the hematopoietic system and circulating in the hemolymph may reach the neurogenic niche and transform into neuronal precursor cells [5], [7], [24]. The current work extends our understanding by defining ultrastructural characteristics of the crayfish niche and its relationship with the vasculature. Evidence presented here supports a connection between the niche and the underlying vasculature, showing that blood vessels, perivascular cells and hemocytes penetrate the retia-like connective tissue complex upon which the niche rests. In addition, fine structural details of resident niche cells indicate specific functions that include regulation of transport from the vascular cavity into the niche. The presence of junctional complexes in Type I cells suggests that if a cell from the vasculature were to migrate into the niche from the vascular cavity, this would potentially involve paracellular or transcellular diapedesis. Therefore, in addition to elaborating our understanding of neurovascular relationships, the evidence presented here suggests hypotheses regarding the mode of cellular addition to the niche, which can be tested in future experiments.
